# Comparison of international guidelines for diagnosis of hepatocellular carcinoma and implications for transplant allocation in liver transplantation candidates with gadoxetic acid enhanced liver MRI versus contrast enhanced CT: a prospective study with liver explant histopathological correlation

**DOI:** 10.1186/s40644-022-00497-9

**Published:** 2022-10-04

**Authors:** Devang Odedra, Ali Babaei Jandaghi, Rajesh Bhayana, Khaled Y. Elbanna, Osvaldo Espin-Garcia, Sandra E. Fischer, Anand Ghanekar, Gonzalo Sapisochin, Kartik S. Jhaveri

**Affiliations:** 1 Department of Medical Imaging, University Health Network Mount Sinai Hospital and Women’s College Hospital, Toronto, ON Canada; 2 Department of Medical Imaging, University Health Network Mount Sinai Hospital and Women’s College Hospital University of Toronto, Toronto, ON Canada; 3 Department of Biostatistics, Princess Margaret Cancer Centre, University Health Network, Toronto, ON Canada; 4 Department of Epidemiology and Biostatistics, Western University, London, Canada; 5 Department of Laboratory Medicine and Pathobiology, University of Toronto, Toronto, ON Canada; 6 Department of Pathology, University Health Network and University of Toronto, Toronto, ON Canada; 7 Department of Surgery, University Health Network and Toronto General Hospital University of Toronto, Toronto, ON Canada

**Keywords:** Hepatocellular carcinoma, Gadoxetic acid enhanced magnetic resonance imaging, Liver imaging and data reporting system, Liver transplantation, Milan criteria

## Abstract

**Objectives:**

To compare the diagnostic performance of international hepatocellular carcinoma (HCC) guidelines with gadoxetic acid-enhanced MRI (EOB-MRI) and contrast-enhanced Computed tomography (CECT) and their impact on liver transplant (LT) allocation in cirrhotic patients with explant histopathology correlation.

**Methods:**

In this prospective single-centre ethics-approved study, 101 cirrhotic patients were consecutively enrolled with informed consent from the pre-LT clinic. They underwent CECT and EOB-MRI alternately at three monthly intervals until LT or removal from LT list. Two abdominal radiologists, blinded to explant histopathology, independently recorded liver lesions visible on CECT and EOB-MRI. Imaging-based HCC scores were assigned to non-treated liver lesions utilizing Liver Imaging Reporting and Data System (LI-RADS), European Association for the Study of the Liver (EASL), Asian-Pacific Association for the Study of the Liver (APASL) and Korean Liver Cancer Association-National Cancer Center (KLCA) guidelines. Liver explant histopathology was the reference standard. Simulated LT eligibility was assessed as per Milan criteria (MC) in reference to explant histopathology.

**Results:**

One hundred and three non-treated HCC and 12 non-HCC malignancy were identified at explant histopathology in 34 patients (29 men, 5 women, age 55–73 years). Higher HCC sensitivities of statistical significance were observed with EOB-MRI for LI-RADS 4 + 5, APASL and KLCA compared to LI-RADS 5 and EASL with greatest sensitivity obtained for LIRADS 4 + 5 lesions. HCC sensitivities by all guidelines with both EOB-MRI and CECT were significantly lower if all histopathology-detected HCCs were included in the analysis, compared to imaging-visible lesions only. A significantly greater variation in HCC sensitivity was noted across the guidelines with EOB-MRI compared to CECT. No significant differences in simulated LT eligibility based on MC were observed across the HCC scoring guidelines with EOB-MRI or CECT.

**Conclusion:**

HCC sensitivities are variable depending on scoring guideline, lesion size and imaging modality utilised. Prior studies that included only lesions visible on pre-operative imaging overestimate the diagnostic performance of HCC scoring guidelines. Per-lesion differences in HCC diagnosis across these guidelines did not impact patient-level LT eligibility based on MC.

**Supplementary Information:**

The online version contains supplementary material available at 10.1186/s40644-022-00497-9.

## Introduction

Several international scientific societies focused on liver diseases have developed guidelines for the diagnosis and management of HCC [1–4]. While these guidelines share the core features for imaging diagnosis of HCC, there are differences therein stemming from locoregional liver disease prevalence and treatment strategies [5]. In Eastern countries, where the prevalence of hepatitis B and C is higher, the guidelines are geared towards achieving high sensitivity for early detection of HCC with focus on locoregional therapies such as ablation or resection. Whilst, in western countries, where the prevalence of alcoholic liver disease and fatty liver disease is greater, the treatment strategies are broadly divided across locoregional, systematic therapies as well as liver transplantation (LT). Western guidelines are also designed for providing a high imaging specificity for HCC diagnosis, thereby obviating the need for biopsy [2, 6, 7].

Imaging also plays a critical role in determining the LT eligibility in HCC patients with several criteria developed across the world for optimizing LT allocation. The Milan Criteria (MC), based on the size and number of HCC diagnosed by imaging, have been well-studied and demonstrated to be effective for this purpose [8]. This further signifies the need to evaluate the impact of international guidelines on HCC diagnosis and LT allocation.

Several prior studies have compared various international HCC scoring guidelines [5, 7, 9–11]. However, many of these are retrospective, focused on Asian population or utilized a non-histopathological reference standard. In this prospective study with explant histopathology correlation, we aimed to compare four international guidelines for HCC diagnosis and their potential impact on LT eligibility with gadoxetic acid enhanced liver MRI (EOB-MRI) and contrast-enhanced computed tomography (CECT). Specifically, we evaluated representative guidelines from both the Eastern and Western scientific societies, namely the American Association for the Study of Liver Disease (AASLD)/Liver Imaging Reporting and Data System (LI-RADS), European Association for the Study of the Liver (EASL), Asian-Pacific Association for the study of the Liver (APASL) and Korean Liver Cancer Association-National Cancer Center (KLCA) [1–4]

## Methods

### Study participants

This was a prospective, single-institution HIPAA compliant Institutional Ethics Board approved study. The patients were enrolled consecutively after informed consent between November 2017 and April 2021, as per inclusion criteria: (a) liver cirrhosis (b) enlisted for LT with a high probability of undergoing transplantation within 12 months (c) diagnosed or suspected HCC with priority Model for End-stage Liver Disease (MELD) score. Exclusion criteria were: patient age less than 18 years, low glomerular filtration rate (GFR) (< 30 mL/min/1.73 m2), high total bilirubin (> 3 mg/dl), pregnancy, contraindications to EOB-MRI such as pacemaker or a ferromagnetic implant, history of contrast allergy, previous radiation, local or systemic treatment for HCC, and those delisted from the transplant list. The imaging alternated between EOB-MRI and CECT at three monthly intervals until LT.

### Imaging techniques

CECT was performed using two scanners (Aquilion ONE or Aquilion 64, Toshiba CA, USA;). CECT protocol and parameters are summarized in (Supplementary Table 1**)**. Patients also underwent EOB-MRI with a 1.5 T (Magnetom Avanto; Siemens Healthcare, Erlangen, Germany) or 3 T (Magnetom Verio with Tim system; Siemens Health care, Erlangen, Germany) MRI scanner with multichannel phased array coils (16 or 32 channels) using a standard liver EOB-MRI protocol (Supplementary Table 2). Gadoxetic acid (Primovist or Eovist, Bayer AG, Germany) was administered intravenously with a power injector (Medrad® Spectris Solaris® EP MR Injection system, Bayer Healthcare, Whippany, USA).

### Imaging evaluation

EOB-MRI and CECT performed closest to the LT were retrieved and anonymized for review from the departmental Picture Archiving and Communication System (PACS). Two faculty abdominal radiologists, with 5 and 3 years of experience independently reviewed the images. They were blinded to the explant histopathology results but were aware that the cohort comprised of cirrhotic patients enlisted for LT.

The readers evaluated all included EOB-MRI and CECT and characterized non-treated liver lesions using LI-RADS version 2018 (Supplementary Table 3) including the presence or absence of major and ancillary imaging features. For each liver lesion, the size; location (based on Couinaud segmental anatomy); signal intensities on pre-contrast T1-weighted, T2-weighted, post contrast T1-weighted dynamic and hepatobiliary phase images; pattern of enhancement and washout (peripheral vs non-peripheral); presence of restricted diffusion; intralesional fat, iron or blood products; enhancing or non-enhancing capsule; and nodule-in-nodule or mosaic architectures were recorded. The maximum lesion diameter was measured on the hepatobiliary phase or portal venous phase (PVP), and if invisible on both of these phases, it was measured on the sequence that best demonstrated the lesion margins. Subtraction images were reviewed to evaluate arterial phase hyperenhancement (APHE). Washout and capsule enhancement were determined on PVP of EOB-MRI and on PVP or equilibrium phase on CECT. Threshold growth could not be included since the imaging analysis was based on single time point examinations with no comparisons to prior examinations.

### HCC score assignment

**Fig. 1:**
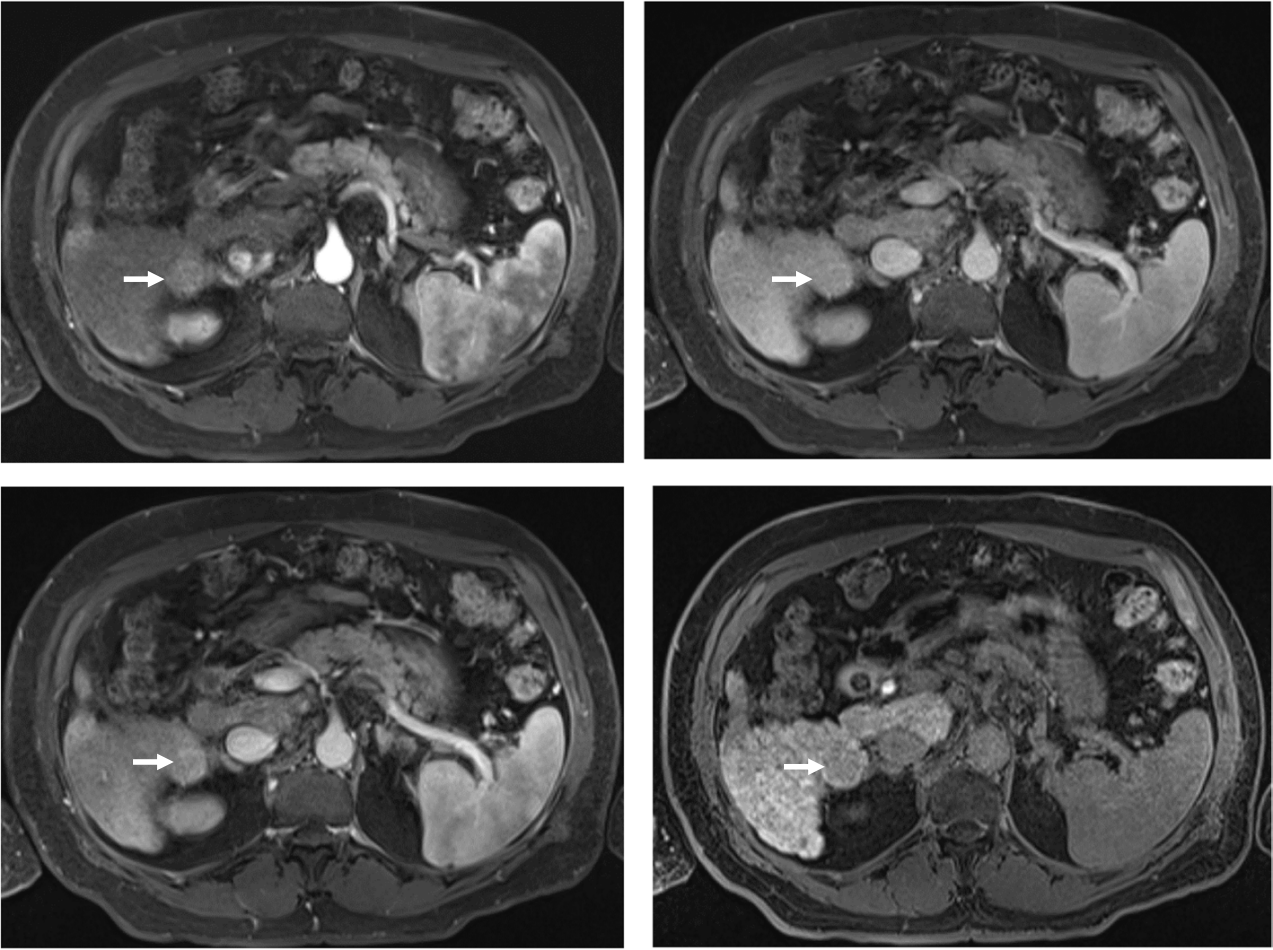
A 70-year-old male with chronic hepatitis B-related cirrhosis. Axial T1-weighted post-gadolinium fat-saturated images demonstrate a segment 6, 27 mm lesion with arterial-phase hyperenhancement (**A**), no washout on portal venous (**B**) or hepatic venous (**C**) phases, and hypointensity on hepatobiliary phase (**D**). The lesion would not count as having a washout as per LI-RADS (LI-RADS 4) and EASL (non-HCC) guidelines but would be considered to have washout as per APASL and KLCA guidelines (HCC as per both guidelines)

**Fig. 2:**
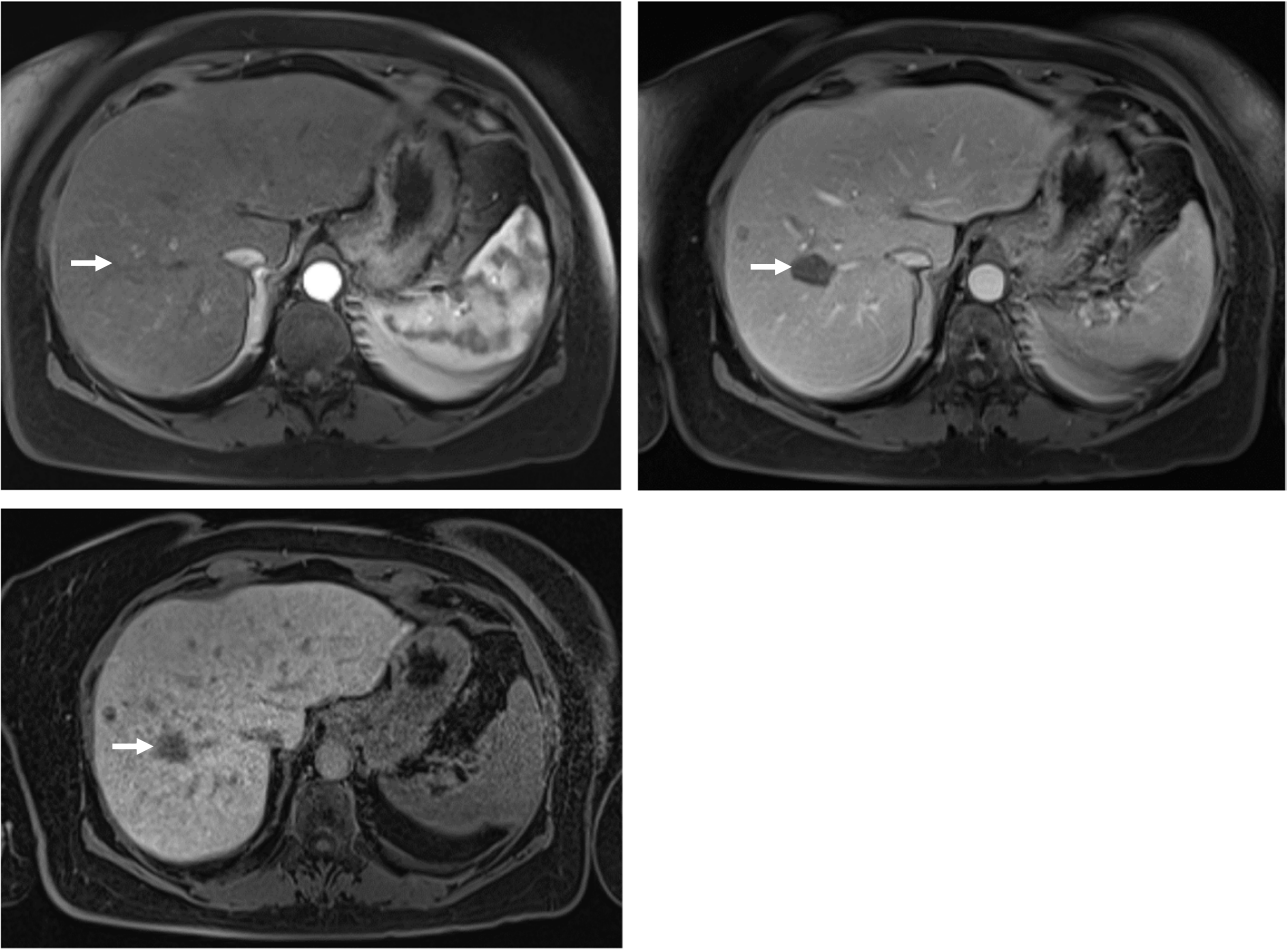
A 64-year-old female with non-alcoholic steatohepatitis. Axial T1-weighted post-gadolinium fat-saturated images demonstrate a segment 8, 25 mm lesion with no arterial phase hyperenhancement (APHE) (**A**), but with washout on portal venous phase (**B**) and hypointensity on hepatobiliary phase (**C**). Despite of the washout, the lesion would not be in keeping with HCC as per EASL, APASL and KLCA due to lack of APHE. As per LI-RADS, however, the lesion can be categorized as LI-RADS 4

HCC scores were assigned to all recorded non-treated liver lesions based on characteristics documented by each reader in accordance with the published international guidelines from LI-RADS, EASL, APASL and KLCA [12]. Major differences between the guidelines are shown in Table 1 (case examples in Figs. 1 and 2). For LI-RADS, separate analyses were performed for LI-RADS 5 only and combined LI-RADS 4 + 5 lesions.

**Table 1:** Major differences in the scoring guidelines

Lesion Size	AASLD/LI-RADS	EASL	APASL	KLCA-NCC
	Any	> 1 cm	Any	> 1 cm
Criteria for diagnosis of HCC	LR-5 (i) Lesion size ≥ 20 mm APHE and one of the following: - Non-peripheral washout - Enhancing capsule - Threshold growth (ii) Lesion size 10–19 mm and 1 of the following - Non peripheral washout - threshold growth (iii) Lesion size 10–19 mm and 2 or more of the following - Non peripheral washout - Threshold growth - Enhancing capsule	APHE and Washout	APHE and washout	APHE and washout
Phases accepted for washout	PVP or HVP (Extracellular agent) PVP only (EOB-MRI)	PVP only	PVP or HBP	PVP or TP or HBP

### Histopathology

All the liver explants were examined by a pathologist with more than 15 years of experience in hepatobiliary disease. The explant livers were sectioned in to 5-mm thick slices in the sagittal plane, and all the suspicious macroscopic nodules at gross examination, any bulging nodule and any lesion macroscopically different in color compared to intervening liver, underwent histopathological evaluation. Nodule size was measured on gross examination. If a lesion detected on pre-operative imaging could not be identified on the sections, further thinner slices were cut from the corresponding regions. Final histopathological assessment included diagnosis, tumour size, segment location, degree of tumour differentiation, presence of microvascular or macrovascular invasion, presence of capsule and degree of necrosis. Tumour staging was reported according to the 8^th^ edition staging system of the American Joint Committee on Cancer (AJCC) [13]. All liver explants were included in the study, regardless of the number of lesions observed per explant.

### Radiology-pathology correlation

A study investigator correlated the non-treated liver lesions recorded by each reader on EOB-MRI and CECT with those seen at explant pathology by matching lesion size and liver segment location. Lesions were considered to match if the difference between histologically and radiologically measured size was less than 10 mm, and no other lesions of similar size were seen in the vicinity. Lesions were grouped into < 1 cm and > 1 cm in size for analysis. Cholangiocarcinoma and mixed HCC-cholangiocarcinoma tumors were considered non-HCC tumors. Two separate analyses were performed (i) A “real-world scenario” (i.e., “All lesions”) wherein all lesions detected on histopathology were included and those HCCs not seen on imaging were categorized as false-negatives (FN) or (ii) A “matched scenario” (i.e., “Imaging-visible lesions only”). wherein only the lesions detected on imaging with corresponding histopathology correlation were included for analysis.

### Liver transplant allocation

At our institution, prospective LT allocation is determined as per Extended Toronto Criteria (ETC), which offers LT irrespective of HCC size or number but requires absence of: macrovascular invasion, extrahepatic disease, systemic cancer-related symptoms and poor tumor differentiation [14]. Thus, we simulated LT eligibility using the MC since it is also the more widely utilized criteria. LT eligibility as per MC was assessed based on preoperative imaging-based HCC diagnosis as per the four international guidelines referenced against explant histopathology. The MC were met if there was (a) a single HCC ≤ 5 cm or (b) 3 or less HCCs ≤ 3 cm, with absence of (c) vascular invasion, and (d) extrahepatic metastatic disease [15].

### Statistical analysis

The diagnostic performance analysis was performed on a per-lesion basis while LT eligibility was assessed at a patient level. The diagnostic performances and accuracies of LT eligibility by HCC guidelines were compared with a test for two proportions. Correction for multiple testing across pairwise comparisons was calculated using the Holm’s method. The degree of variability in the diagnostic performance of CECT and EOB-MRI across the guidelines was compared with Levene’s test. A *p*-value (or q-value for multiple comparisons) of less than 0.05 was considered statistically significant. Inter-reader concordance was computed as per pairwise unweighted kappa coefficients.

## Results

### Patient Demographics and Liver lesions (Table 2)

**Table 2:** Patient demographics and liver lesion characteristics

Total Number of Patients	34
Age of patients, average (range)	66.2 (55.2–73.4)
Sex	29 (M), 5 (F)
Cause of Liver Disease	
HCV	14
HBV	6
Alcohol	7
NASH	6
Other	1
Type of Liver Transplantation	
Living Donor	5
Deceased Donor	29
Total number of lesions at histopathology	115
Number of lesions per patient, range	1 – 15
Size of the lesion, mm, average (range)	12.2 (2–35)
< 1 cm	43
> 1 cm	72
Histopathology	
HCC	103
HCC + CC	2
CC	1
Benign	9

*HCV* Hepatitis C, *HBV* Hepatitis B, *NASH* Non-alcoholic steatohepatitis, *CC* Cholangiocarcinoma

**Fig. 3:**
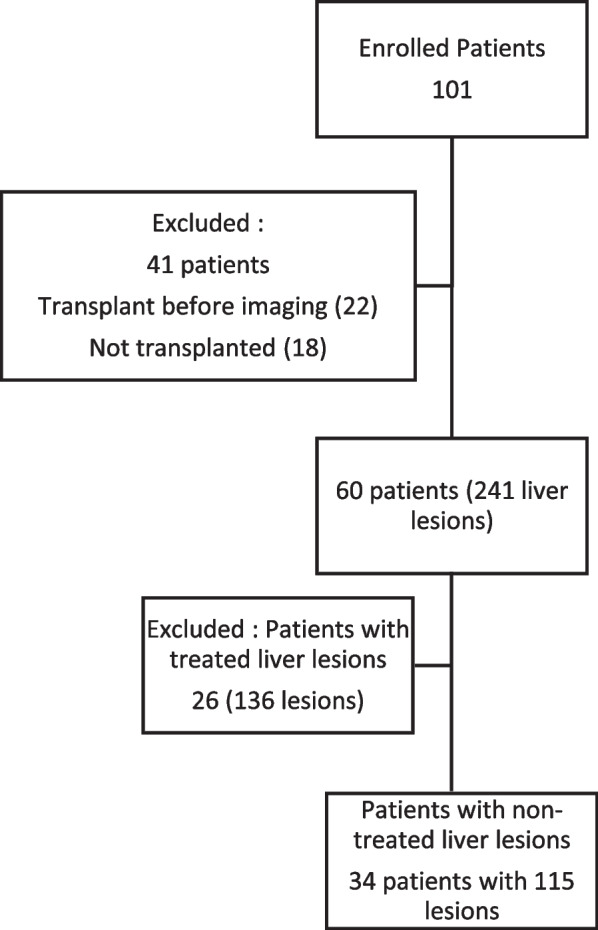
Patient enrollment and study flowchart

The study cohort comprised of 101 enrolled patients, from which 60 patients with 241 liver lesions underwent LT. 26 patients with 136 liver lesions were excluded due to previous HCC therapy. The final patient cohort **(**Fig. 3**)** comprised of 115 non-treated liver lesions in 34 patients with a mean age of 66 years (age range 55–73 years). Chronic HCV was the most common cause of liver disease. Majority of patients underwent deceased donor transplant. A total of 115 liver lesions (103 HCC, 3 non-HCC malignancy and 9 benign lesions) were noted at histopathology, with per-patient lesion count of 1 to 15 and lesion size range of 2 to 35 mm. Reader 1 (R1) and Reader 2 (R2) identified 78 and 80 lesions, respectively, on EOB-MRI while 46 and 42 lesions were identified, respectively, on CECT.

### Diagnostic Performance of Scoring Guidelines with EOB-MRI (Table 3)

**Table 3:** HCC Sensitivity and Specificity with EOB-MRI for various guidelines. (Numbers represent Sensitivity/Specificity in percentages)

	** > 1 cm, all lesions**		** > 1 cm, imaging visible lesions only**		**All sizes, imaging visible lesions only**		**All sizes, all lesions**	
	Reader 1	Reader 2	Reader 1	Reader 2	Reader 1	Reader 2	Reader 1	**Reader 2**
**LI-RADS 4 + 5**	63.1/91.7	52.8/91.7	98.1/83.3	81.0/80.0	95.9/83.3	75.0/80.0	68.0/91.7	52.4/91.7
**LI-RADS 5 only**	35.7/100.0	32.6/91.7	55.6/100.0	51.7/80.0	41.1/100.0	40.3/80.0	29.1/100.0	28.2/91.7
**EASL**	32.1/100.0	32.6/91.7	50.0/100.0	50.0/80.0	37.0/100.0	40.3/80.0	26.2/100.0	28.2/91.7
**APASL**	59.5/100.0	51.7/75.0	92.6/83.3	79.3/80.0	89.0/83.3	73.6/80.0	63.1/100.0	51.5/75.0
**KLCA**	60.7/91.7	51.7/75.0	94.4/83.3	79.3/80.0	69.9/83.3	63.9/80.0	49.5/91.7	44.7/75.0

The sensitivity for HCC diagnosis varied based on the HCC guideline, lesion size and visibility on imaging in reference to histopathology. Overall, significantly higher sensitivities were realised for both readers in the matched scenario compared to the real-world scenario.

In reference to all lesions > 1 cm on histopathology, the sensitivity for HCC diagnosis was significantly higher by LI-RADS 4 + 5 (63.1%), KLCA (60.7%) and APASL (59.5%) compared with LI-RADS 5 (35.7%) and EASL (32.1%) for R1. Similar trends were observed for R2, albeit without statistical significance. In reference to only imaging-visible lesions > 1 cm, LI-RADS 4 + 5 again had the highest sensitivity (98.1/81.0% for R1/R2), followed by KLCA (94.4/79.3% for R1/R2) and APASL (92.6/79.3% for R1/R2) with each demonstrating statistically significant differences versus EASL and LI-RADS 5 (Supplementary Table 4).

In reference to all lesions noted on histopathology regardless of size, similar trends were observed. LI-RADS 4 + 5 again demonstrated the greatest HCC sensitivity (68.0/52.4% for R1/R2) with statistically significant differences against LI-RADS 5 (29.1/28.2% for R1/R2) and EASL (26.2/28.2% for R1/R2). The next highest sensitivities for R1 were observed in APASL (63.1%) and KLCA (49.5%), which were significantly higher than LI-RADS 5 and EASL. For R2, similar trends were observed except that the differences between KLCA and, LI-RADS 5 and EASL, were not statistically significant. In reference to imaging-visible lesions only regardless of size, R1 had significantly higher HCC sensitivity for LI-RADS 4 + 5 (95.9%) compared to LI-RADS 5 (41.1%), EASL (37.0%) and KLCA (69.9%). LI-RADS 4 + 5 (75.0%) also demonstrated the highest sensitivity for R2, with statistically significant differences noted against LI-RADS 5 (40.3%) and EASL (40.3%). The next highest sensitivities in R1 were noted with APASL (89.0%) and KLCA (69.9%), which were significantly higher compared to LI-RADS 5 and EASL. APASL demonstrated higher sensitivity than KLCA (89.0% vs 69.9%, *p* = 0.047). Similar trends were observed with R2, with statistically significant differences between APASL (73.6%), and LI-RADS 5 (40.3%) and EASL (40.3%). KLCA did not demonstrate statistical significance against other guidelines (Supplementary Table 5).

The specificity for HCC across the guidelines varied 83.3–100% for R1 and 75.0–100% for R2. LI-RADS 5 and EASL provided a consistent specificity of 100% for R1, regardless of lesion size or visibility on imaging. The specificity for LI-RADS 5 and EASL for R2 ranged 80.0–91.7%. APASL and KLCA demonstrated the lowest specificity, ranging 83.3–100% for R1 and 75.0–80.0% for R2. The differences between specificities across the guidelines were not statistically significant.

### Diagnostic Performance of Scoring Guidelines with CECT (Table 4)

**Table 4:** HCC Sensitivity and Specificity with CECT for various guidelines. (Numbers represent Sensitivity/Specificity in percentages)

	** > 1 cm, all lesions**		** > 1 cm, imaging visible lesions only**		**All sizes, imaging visible lesions only**		**All sizes, all lesions**	
	Reader 1	Reader 2	Reader 1	Reader 1	Reader 1	Reader 2	Reader 1	Reader 2
**LI-RADS 4 + 5**	28.0/100	21.9/100	81.2/100	67.7/100	76.2/100	63.2/100	31.1/100	23.3/100
**LI-RADS 5 only**	28.0/100	19.8/100	81.2/100	61.3/100	61.9/100	50.0/100	25.2/100	18.4/100
**EASL**	15.1/100	18.8/100	43.8/100	58.1/100	33.3/100	47.4/100	13.6/100	17.5/100
**APASL**	28.0/100	19.8/100	81.2/100	61.3/100	76.2/100	57.9/100	31.1/100	21.4/100
**KLCA**	28.0/100	19.8/100	81.2/100	61.3/100	61.9/100	50.0/100	25.2/100	18.4/100

The HCC sensitivities obtained by CECT with the scoring guidelines were generally lower compared to those with EOB-MRI. Similar to EOB-MRI, the diagnostic performance was higher overall for the matched-scenario compared to the real-world scenario.

In reference to all lesions > 1 cm, EASL showed the lowest sensitivity (15.1%) for R1 compared to the other guidelines, which all had the same sensitivity (28.0%). For R2, EASL again showed the lowest sensitivity (18.8%), while LI-RADS 4 + 5 demonstrated the highest sensitivity at 21.9%. If only imaging-visible lesions > 1 cm were included in the analysis, the sensitivity for R1 increased to 43.8% with EASL and 81.2% for the remaining guidelines. For R2, the sensitivity improved to 58.1% with EASL, which again remained the lowest, and 67.7% with LI-RADS 4 + 5, which remained the highest (Supplementary table 6).

For all lesions regardless of size, R1 had the lowest sensitivity with EASL (13.6%) and the highest sensitivities with LI-RADS 4 + 5 and APASL (31.1%). A similar trend was noted for R2 with EASL being the lowest (17.5%) and LI-RADS 4 + 5 being the highest (23.3%). If only imaging-visible lesions were included, for R1 the HCC sensitivity improved to 33.3% with EASL, still persisting as the lowest while increasing to 76.2% with LI-RADS 4 + 5 and APASL, which maintained the highest positions. For R2, the sensitivities improved to 47.4% with EASL and 63.2% with LI-RADS 4 + 5. Statistically significant differences in HCC sensitivities were observed for R1 in the imaging-visible lesions of all sizes between EASL, APASL and LI-RADS 4 + 5. No additional statistically significant trends were derived amongst the HCC sensitivities with CECT (Supplementary table 7).

The specificity for CECT was 100% with both readers for all the scoring guidelines, regardless of size cut-off or visibility on imaging. This was generally higher than EOB-MRI although there were no statistically significant differences across the guidelines.

### Variation in HCC diagnosis between guidelines with EOB-MRI and CECT (Table 5)

**Table 5:** Comparison of variances in HCC sensitivity and specificity across guidelines with EOB-MRI and CECT using Levene’s test

LESION TYPE		SENSITIVITY			SPECIFICITY		
		***σ***_***MR***_	***σ***_***CT***_	***p*** **-Value**	***σ***_***MR***_	***σ***_***CT***_	***p*** **-Value**
** > 1 cm All Path HCC**	Reader #1	0.0140	0.053	**0.037**	0.044	0	** < 10** ^**–3**^
	Reader #2	0.099	0.010	**0.011**	0.088	0	**0.010**
** > 1 cm imaging visible only**	Reader #1	0.216	0.151	0.347	0.088	0	**0.010**
	Reader #2	0.148	0.033	**0.015**	0.207	0	**0.010**
**All size All Path**	Reader #1	0.183	0.066	**0.019**	0.044	0	** < 10** ^**–3**^
	Reader #2	0.115	0.023	**0.011**	0.088	0	**0.010**
**All size imaging visible only**	Reader #1	0.258	0.167	0.179	0.088	0	**0.010**
	Reader #2	0.169	0.062	**0.018**	0.207	0	**0.010**

*σ*_*MR*_: EOB-MR standard deviation, σ_*CT*_: CECT Standard deviation

The standard deviations of HCC sensitivity and specificity with EOB-MRI and CECT across the various guidelines were compared for both readers. In terms of sensitivity, R2 demonstrated significantly greater standard deviation across the guidelines with EOB-MRI compared to CECT, regardless of lesion size or imaging visibility. Similar trend was observed for R1, with the exception of the imaging visible lesions > 1 cm and all lesions regardless of size groups wherein no statistically significant differences were noted. In terms of specificity, there was no variation across guidelines with CECT and all the standard deviations were significantly greater with EOB-MRI compared to CECT.

### Transplant Allocation (Table 6)

**Table 6:** Accuracy for simulated LT eligibility as per Milan criteria with EOB-MRI and CECT versus explant histopathology. (Numbers represent accuracy in percentages)

	**EOB-MRI**		**CECT**	
	Reader 1	Reader 2	Reader 1	Reader 2
**LI-RADS 4 + 5**	82.4	82.4	76.5	79.4
**LI-RADS 5**	94.1	85.3	79.4	82.4
**EASL**	85.3	82.4	73.5	79.4
**APASL**	88.2	85.3	76.5	82.4
**KLCA**	85.3	82.4	76.5	79.4

The accuracy for simulated transplant eligibility based on MC across the HCC guidelines ranged between 82.4 – 94.1% with EOB-MRI and 73.5 – 79.4% with CECT for R1. For R2, the accuracy ranged 82.4 – 85.3% with EOB-MRI and 79.4 – 82.4% with CECT. Although there were no statistically significant differences in LT eligibility accuracies based on the HCC guidelines with both imaging modalities and either reader, the highest accuracy was obtained for LIRADS 5 for R1 with both EOB-MRI and CECT (94.1% and 79.4%, respectively). In comparison, R2 obtained highest accuracy for LI-RADS 5 and APASL with both EOB-MRI and CECT (85.3% and 82.4%, respectively).

### Inter-reader agreement (Table 7)

**Table 7:** Inter-reader agreements in terms of Kappa values

Guideline	EOB-MRI	CECT
LI-RADS	0.36	0.54
EASL	0.30	0.63
APASL	0.43	0.62
KLCA	0.32	0.70

The inter-reader concordance between the two readers across various HCC guidelines ranged from fair to moderate (k = 0.30–0.43) for EOB-MRI and moderate to good (k = 0.54–0.70) for CECT [16].

## Discussion

In this prospective study, we assessed the impact of various international guidelines on HCC diagnosis and LT eligibility with EOB-MRI and CECT in reference to explant histopathology. We addressed some of the key shortcomings of prior studies through prospective explant histopathological correlation in the entire included patient cohort. Additionally, we did not exclude any patients based on liver lesion size, number, or invisibility on pre-operative imaging, with the specific aim of providing results for a real-world scenario.

Overall, regardless of lesion size and visibility on imaging, we observed the highest sensitivities for HCC on EOB-MRI for LI-RADS 4 + 5, KLCA and APASL respectively, which were all greater than LI-RADS 5 and EASL. The inclusion of LI-RADS 4 category as HCC, not surprisingly, improved the sensitivity with EOB-MRI likely secondary to inclusion of ancillary features. Particularly, the inclusion of hepatobiliary phase hypointensity, which expands the washout definition similar to APASL and KLCA guidelines, likely played a major role [2]. In contrast, the differences in sensitivities between LI-RADS 4 + 5 and LI-RADS 5 with CECT were not as pronounced as with EOB-MRI, likely secondary to fewer ancillary features available with CECT for upstaging [17]. Prior studies, which included only LI-RADS 5 category as “definitely HCC”, have also observed lower sensitivities compared with APASL and EASL [7, 10, 11]. In this study, we simulated a real-world scenario, wherein all HCCs seen at explant histopathology were included in the analysis and those without pre-operative imaging correlate were considered as false negatives. This resulted in a significant drop in HCC sensitivity by almost one-third for EOB-MRI and approximately one-half for CECT across the guidelines. In many of the prior relevant studies, the lesions not seen prospectively on pre-operative imaging were excluded, which may have overestimated the diagnostic performance of the guidelines [10, 11]. In contrast to sensitivity, the imaging-visibility of the lesions did not make a significant difference in the specificities for both EOB-MRI and CECT, likely due to a small sample size and low count of non-HCC malignancies in our study.

Two prior studies, by Jeon et al. and Clarke et al., investigated the diagnostic performance of various guidelines on EOB-MRI, with explant histopathological correlation [7, 18]. In contrast to our study, the study by Jeon et al. was retrospective in design and only included lesions seen on pre-operative imaging. Additionally, the lesions were marked by arrows by one of the authors after radiology-pathology correlation prior to assessment by the reviewers which can be considered significant bias and not a true estimation of HCC sensitivity. They reported highest HCC sensitivity with APASL and KLCA, which is in line with our results if we only considered LI-RADS 5 as definite HCC. Further, the highest specificity was observed for LI-RADS, which is also similar to our results. The study by Clarke et al. was also retrospective in design and included only EOB-MRI visible lesions ≥ 1 cm. The authors observed similar trends, noting higher sensitivities with Eastern guidelines from the Japan Society of Hepatology (JSH) and APASL. Conversely, the Western guidelines including LI-RADS, Organ Procurement and Transplantation Network (OPTN) and EASL produced the highest specificity. Of note, the study did not report on statistical differences between the diagnostic performance of the guidelines. A study by Seo et al. compared the diagnostic performance of several international guidelines with CECT for LT candidates with explant correlation, as well as LT allocation based on MC [19]. This study was also retrospective in design with inclusion again of only lesions seen on CECT. Furthermore, the guidelines used in that study (LI-RADS 2014) have since been significantly updated since, limiting direct comparison with our study. However, the HCC sensitivities observed in our study for imaging-visible lesions are overall comparable to previously reported values [14, 20].

We observed greater variances in the HCC sensitivities across guidelines with EOB-MRI compared to CECT. This was particularly evident in all lesion categories regardless of size and visibility on imaging for one of the readers. This is also concordant with the lack of statistically significant differences in HCC sensitivities across the guidelines with CECT compared to EOB-MRI. We suspect that the increased variability observed with EOB-MRI may be partly related to the higher number of imaging sequences and lesion characteristics being assessed, further compounded by greater inter-reader differences. Thus, we surmise that the differences in HCC diagnosis across various guidelines are further accentuated with EOB-MRI compared to CECT. This has not been adequately investigated or reported in previous studies.

In our study, while there were significant differences in HCC sensitivities and specificities between EOB-MRI and CECT across the guidelines at a per-lesion level, this did not translate into statistically significant differences for per-patient LT eligibility as per MC. While LI-RADS had higher accuracies for simulated LT eligibility with both readers, the differences were not statistically significant compared to other guidelines. This contrasts the results of Jeon et al., who observed the highest accuracy for unsuitable LT candidates with KLCA guidelines based on MC [7]. Seo et al. also investigated the accuracy of CECT for LT allocation based on MC and found overall good accuracy with no significant differences across the guidelines. Thus, imaging-based LT allocation as per MC appears to perform similarly regardless of the HCC guideline utilized. We do recognize that MC is not the only clinical decision tool available for LT allocation. Of note, at our institution, a more patient-tailored approach to transplant allocation is utilized that incorporates the patient’s cancer-related symptoms and the degree of tumour differentiation [21].

Our study has several limitations. Our study sample size was small, limiting strong inference of results. However, this study was prospective and included explant histopathology correlation for the entire study cohort. The study population only consisted of cirrhotic patients on LT list. This may introduce a selection bias and limit the applicability of the study to only LT candidates, which is still of value in our opinion. Furthermore, the prospective imaging and histopathological assessment were focused primarily on HCC detection and diagnosis. Hence, benign lesions and non-HCC malignancies were possibly underrepresented in the study population, limiting the implication of specificity results. Future studies with inclusion of more benign lesions would be helpful in the assessment of specificities obtained amongst the various guidelines. Additionally, we calculated the HCC scores based on only a single timepoint without the use of threshold growth, which plays an important role in LI-RADS. This may have underestimated the sensitivity of LI-RADS in our study. Furthermore, there was interobserver variability amongst the readers particularly with EOB-MRI, which likely had an impact on the variances observed across the different guidelines. Finally, the number of lesions detected by CECT were significantly lower than EOB-MRI, and while this applied to both readers, it should be factored into the interpretation of overall results.

## Conclusion

In conclusion, highest sensitivities for HCC diagnosis by EOB-MRI in cirrhotic patients listed for LT were observed with LI-RADS 4 + 5, APASL and KLCA. True sensitivities suffered significantly if all HCCs identified on histopathology were included regardless of their visibility on pre-operative imaging. This is reflective of the real-world scenario and suggests that prior literature, primarily focused on imaging-visible lesions only, may have overestimated the diagnostic performance of HCC guidelines. Further, the utilization of EOB-MRI may accentuate the differences in HCC diagnosis across various guidelines. However, at a patient level, the differences in HCC diagnosis due to various guidelines do not significantly impact the accuracies for LT eligibility as per MC with EOB-MRI and CECT.

## Supplementary Information

**Additional file 1: Supplementary Table 1.** CECT quadriphasic liver protocol (Aquilion 64). **Supplementary Table 2.** Protocol for gadoxetic acid-enhanced liver MRI (Gd-EOB-MRI). **Supplementary Table 3.** LI-RADS v2018 – Major and Ancillary Features with EOB-MRI. **Supplementary Table 4.** Differences between sensitivities for scoring guidelines for EOB-MRI for lesions > 1 cm (numbers represent p values, all lesions seen on histopathology/ imaging-visible lesions only). **SupplementaryTable 5.** Differences between sensitivities for scoring guidelines systems forEOB-MRI for lesions of all sizes (numbers represent p values, all lesions seenon histopathology/imaging visible lesions only). **Supplementary Table 6.** Differences between sensitivities for scoring guidelines for CECT all lesions size > 1 cm (numbers represent p values, all lesions seen on histopathology/imaging visible lesions only). **Supplementary Table 7.** Differences between sensitivities for scoring guidelines for CECT for lesions of all sizes (numbers represent p values, all lesions seen on histopathology/imaging-visible lesions only).

## Data Availability

The datasets used and/or analysed during the current study are available from the corresponding author on reasonable request.

## Abbreviations

AASLD: American Association for the study of the Liver Disease
AJCC: American Joint Committee on Cancer
APASL: Asian-Pacific Association for the Study of the Liver
CECT: Contrast Enhanced Computed Tomography
EASL: European Association for the Study of the Liver
EOB-MRI: Gadoxetic acid-enhanced MRI
FN: False Negative
HCC: Hepatocellular Carcinoma
KLCA: Korean Liver Cancer Association – National Cancer Center
LI-RADS: Liver imaging reporting and data system
LT: Liver Transplant
MC: Milan Criteria
MELD: Model for End-stage Liver Disease
PACS: Picture Archiving Communicating System
R1: Reader 1
R2: Reader 2
