# Supplementary material for: Comparison of international guidelines for diagnosis of hepatocellular carcinoma and implications for transplant allocation in liver transplantation candidates with gadoxetic acid enhanced liver MRI versus contrast enhanced CT: a prospective study with liver explant histopathological correlation
Source: Cancer Imaging. 2022 Oct 4;22:55. doi: 10.1186/s40644-022-00497-9 (PMC9531508; doi:10.1186/s40644-022-00497-9)
Supplement: Supplementary file 1 — Additional file 1: Supplementary Table 1. CECT quadriphasic liver protocol (Aquilion 64). Supplementary Table 2. Protocol for gadoxetic acid-enhanced liver MRI (Gd-EOB-MRI). Supplementary Table 3. LI-RADS v2018 – Major and Ancillary Features with EOB-MRI. Supplementary Table 4. Differences between sensitivities for scoring guidelines for EOB-MRI for lesions > 1 cm (numbers represent p values, all lesions seen on histopathology/ imaging-visible lesions only). SupplementaryTable 5. Differences between sensitivities for scoring guidelines systems forEOB-MRI for lesions of all sizes (numbers represent p values, all lesions seenon histopathology/imaging visible lesions only). Supplementary Table 6. Differences between sensitivities for scoring guidelines for CECT all lesions size > 1 cm (numbers represent p values, all lesions seen on histopathology/imaging visible lesions only). Supplementary Table 7. Differences between sensitivities for scoring guidelines for CECT for lesions of all sizes (numbers represent p values, all lesions seen on histopathology/imaging-visible lesions only). [file 40644_2022_497_MOESM1_ESM.docx]

Supplementary Table 1. CECT quadriphasic liver protocol (Aquilion 64)

| **CT Scanner parameters** |  |
| --- | --- |
| Axial slice thickness | 5 mm |
| Slice interval | 2.5 mm |
| Detector configuration | 64 × 0.5 (32 mm) |
| Tube voltage | 120 kV |
| Automated tube current | 80-440 mA |
| Helical pitch | 53 |
| Pitch factor | 0.828 |
| Tube rotation time | 0.5 s |
| Craniocaudal coverage | Unenhanced, arterial and delayed phases: from top of the diaphragm to bottom of liver  Venous phase: from top of the diaphragm to below symphysis pubis |
| IV Contrast^*^ | Ultravist 370, GE Healthcare, 2ml/kg to a maximum of 150 mL |
| Phase Delay | Phase 1: no delay  Phase 2: Arterial - Sure Start on abdominal aorta at 100 HU plus  25 sec delays  Phase 3: Venous - 100HU plus 60 sec delays  Phase 4: 180 sec delays |
| Multiplanar reformatted images | Coronal and sagittal planes for all phases with a slice thickness of 3 mm |

* Contrast was administered intravenously, through a minimum 20-gauge intravenous catheter inserted into a forearm vein, with a power injector [Medrad® Stellant® Dual Head] at a rate of 5mL/s.

Supplementary Table 2. Protocol for gadoxetic acid-enhanced liver MRI (Gd-EOB-MRI)

| **Image sequence** | **Field strengths** | **TR (ms)** | **TE (ms)** | **FA (D^◦^)** | **FOV (mm)** | **ST (mm)** | **Voxel size (mm)** | **Fat suppression** | **Respiratory control** | |
| --- | --- | --- | --- | --- | --- | --- | --- | --- | --- | --- |
| **Axial T2 HASTE** | **1.5T** | 1200 | 180 | 160 | 370 | 5 | 1.2 x 1.2 x 5.0 | SPAIR | Breath-hold | |
|  | **3T** | 1200 | 181 | 146 | 380 | 5 | 1.2 x 1.2 x 5.0 |  |  |  |
| **Coronal T2 HASTE** | **1.5T** | 1200 | 180 | 160 | 370 | 4 | 1.2 x 1.2 x 4.0 | None | Breath-hold | |
|  | **3T** | 1600 | 180 | 160 | 380 | 4 | 1.2 x 1.2 x 4.0 |  |  |  |
| **Axial T1 VIBE in-out phase** | **1.5T** | 150 | 1.45-2.69 | 70 | 370 | 4 | 0.7 x 0.7 x 4.0 | None | Breath-hold | |
|  | **3T** | 150 | 1.23-2.46 | 70 | 380 | 4 | 0.7 x 0.7 x 4.0 |  |  |  |
| **DWI ep2d diff b100,600** | **1.5T** | 6300 | 63.0 | - | 400 | 5 | 1.0 x1.0 x 5.0 | SPAIR | Off | |
|  | **3T** | 6600 | 68.0 | - | 400 | 5 | 1.0 x 1.0 x 5.0 |  |  |  |
| **Axial T1 VIBE** | **1.5T** | 4.10 | 1.98 | 10 | 370 | 3 | 1.2 x 1.2 x 3.0 | SPAIR | Breath-hold | |
|  | **3T** | 3.60 | 1.75 | 9 | 380 | 3 | 1.2 x 1.2 x 3.0 |  |  |  |
| **Post-contrast imaging*** | | | | | | | | | |  |
| **Axial T1 VIBE**  **(arterial phase)** | **1.5T** | 3.56 | 1.74 | 10 | 380 | 3 | 1.2 x 1.2 x 3.0 | SPAIR | Breath-hold | |
|  | **3T** | 3.60 | 1.75 | 9 | 380 | 3 | 1.2 x 1.2 x 3.0 |  |  |  |
| **Axial T1 VIBE**  **(portal venous phase)** | **1.5T** | 4.10 | 1.98 | 10 | 370 | 3 | 1.2 x 1.2 x 3.0 | SPAIR | Breath-hold | |
|  | **3T** | 3.60 | 1.75 | 9 | 380 | 3 | 1.2 x 1.2 x 3.0 |  |  |  |
| **Axial T1 VIBE**  **(transitional phase)** | **1.5T** | 4.10 | 1.97 | 10 | 380 | 3 | 1.2 x 1.2 x 3.0 | SPAIR | Breath-hold | |
|  | **3T** | 3.60 | 1.75 | 9 | 380 | 3 | 1.2 x 1.2 x 3.0 |  |  |  |
| **Axial T1 VIBE 20 min**  **(hepatobiliary phase)** | **1.5T** | 4.29 | 1.73 | 25 | 370 | 3 | 1.2 x 1.2 x 3.0 | Q-fat | Breath-hold | |
|  | **3T** | 4.29 | 1.82 | 25 | 360 | 3 | 1.1 x 1.1 x 3.0 |  |  |  |
| **Coronal T1 VIBE 20 min** | **1.5T** | 4.39 | 1.67 | 25 | 420 | 3 | 1.3 x 1.3 x 3.0 | Q-fat | Breath-hold | |
|  | **3T** | 4.39 | 1.75 | 25 | 420 | 3 | 1.3 x 1.3 x 3.0 |  |  |  |
| **Axial T1 CAIPI-VIBE 20 min (hepatobiliary phase)** | **1.5T** | 4.29 | 1.74 | 25 | 360 | 3 | 1.1 x 1.1 x 3.0 | Q-fat | Breath-hold | |
|  | **3T** | 4.02 | TE1 = 1.32  TE2 = 2.55 | 9 | 360 | 1.5 | 1.1 x 1.1 x 1.5 |  |  |  |

*Gadoxetic acid (Primovist or Eovist, Bayer AG, Germany) was administered to patients intravenously through a 22-gauge intravenous catheter inserted into a forearm vein with an MR-compatible power injector (Medrad® Spectris Solaris® EP MR Injection system, Bayer Healthcare, Whippany, USA) at a rate of 1 mL/s (0.025 mmol/kg body weight) followed by a 10-mL normal saline chaser at the same rate.

Supplementary Table 3. LI-RADS v2018 – Major and Ancillary Features with EOB-MRI

| **Major features** | Non-rim arterial hyperenhancement, Washout appearance on PVP, Enhancing capsule |
| --- | --- |
| **Ancillary features favoring malignancies in general** | Mild to moderate T2 hyperintensity, HBP hypointensity, Restricted diffusion, |
| **Ancillary features favoring HCCs in particular** | Nodule-in-nodule architecture, Fat in mass, Blood product in mass |
| **Imaging features favoring non-HCC malignancies** | Targetoid dynamic enhancement including AP rim enhancement, peripheral washout on PVP, delayed centripetal enhancement, targetoid TP or HBP appearance, and targetoid restriction |

AP: arterial phase, EOB-MRI: Gadoxetic acid-enhanced MRI, HBP: hepatobiliary phase, PVP: portal venous phase, TP: transitional phase

Supplementary Table 4. Differences between sensitivities for scoring guidelines for EOB-MRI for lesions > 1 cm (numbers represent p values, all lesions seen on histopathology/ imaging-visible lesions only)

|  | R1 LI-RADS 4+5 | R1 LI-RADS 5 | R1 EASL | R1 APASL | R2 LI-RADS 4+5 | R2 LI-RADS 5 | R2 EASL | R2 APASL |
| --- | --- | --- | --- | --- | --- | --- | --- | --- |
| LI-RADS 5 | **0.009/<0.001** |  |  |  | 0.150/**0.023** |  |  |  |
| EASL | **0.002/<0.001** | 1.000/1.000 |  |  | 0.150/**0.013** | 1.000/1.000 |  |  |
| APASL | 1.000/1.000 | **0.030/<0.001** | **0.009/<0.001** |  | 1.000/1.000 | 0.197/**0.034** | 0.197/**0.025** |  |
| KLCA | 1.000/1.000 | 0.001/<0.001 | 0.005/<0.001 | 1.000/1.000 | 1.000/1.000 | 0.197/0.034 | 0.197/0.025 | 1.000/1.000 |

Supplementary Table 5. Differences between sensitivities for scoring guidelines systems for EOB-MRI for lesions of all sizes (numbers represent p values, all lesions seen on histopathology/imaging visible lesions only)

|  | R1 LI-RADS 4+5 | R1 LI-RADS 5 | R1 EASL | R1 APASL | R2 LI-RADS 4+5 | R2 LI-RADS 5 | R2 EASL | R2 APASL |
| --- | --- | --- | --- | --- | --- | --- | --- | --- |
| LI-RADS 5 | **<0.001/<0.001** |  |  |  | **0.010/<0.001** |  |  |  |
| EASL | **<0.001/<0.001** | 1.000/1.000 |  |  | **0.010/<0.001** | 1.000/1.000 |  |  |
| APASL | 1.000/0.839 | **<0.001/<0.001** | **<0.001/<0.001** |  | 1.000/1.000 | **0.014/0.001** | **0.014/0.001** |  |
| KLCA | 0.076/<0.001 | 0.035/0.006 | 0.009/0.001 | 0.407/0.047 | 1.000/1.000 | 0.185/0.068 | 0.185/0.068 | 1.000 |

Supplementary Table 6. Differences between sensitivities for scoring guidelines for CECT all lesions size > 1 cm (numbers represent p values, all lesions seen on histopathology/imaging visible lesions only)

|  | R1 LI-RADS 4+5 | R1 LI-RADS 5 | R1 EASL | R1 APASL | R2 LI-RADS 4+5 | R2 LI-RADS 5 | R2 EASL | R2 APASL |
| --- | --- | --- | --- | --- | --- | --- | --- | --- |
| LI-RADS 5 | 1.000/1.000 |  |  |  | 1.000/1.000 |  |  |  |
| EASL | 0.745/0.068 | 0.745/0.068 |  |  | 1.000/1.000 | 1.000/1.000 |  |  |
| APASL | 1.000/1.000 | 1.000/1.000 | 0.745/0.068 |  | 1.000/1.000 | 1.000/1.000 | 1.000/1.00 |  |
| KLCA | 1.000/1.000 | 1.000/1.000 | 0.745/0.068 | 1.000/1.000 | 1.000/1.000 | 1.000/1.000 | 1.000/1.000 | 1.000/1.000 |

Supplementary Table 7. Differences between sensitivities for scoring guidelines for CECT for lesions of all sizes (numbers represent p values, all lesions seen on histopathology/imaging-visible lesions only)

|  | R1 LI-RADS 4+5 | R1 LI-RADS 5 | R1 EASL | R1 APASL | R2 LI-RADS 4+5 | R2 LI-RADS 5 | R2 EASL | R2 APASL |
| --- | --- | --- | --- | --- | --- | --- | --- | --- |
| LI-RADS 5 | 1.000/1.000 |  |  |  | 1.000/1.000 |  |  |  |
| EASL | 0.067/**0.003** | 0.632/0.195 |  |  | 1.000/1.000 | 1.000/1.000 |  |  |
| APASL | 1.000/1.000 | 1.000/1.000 | 0.067/**0.003** |  | 1.000/1.000 | 1.000/1.000 | 1.000/1.000 |  |
| KLCA | 1.000/1.000 | 1.000/1.000 | 0.632/0.195 | 1.000/1.000 | 1.000/1.000 | 1.000/1.000 | 1.000/1.000 | 1.000/1.000 |
